# Argon-helium knife cryoablation plus programmed cell death protein 1 inhibitor in the treatment of advanced soft tissue sarcomas: there is no evidence of the synergistic effects of this combination therapy

**DOI:** 10.3389/fonc.2023.1185291

**Published:** 2023-09-05

**Authors:** Jiaqiang Wang, Dengwei Zong, Shuping Dong, Shilei Gao, Yonghao Yang, Peng Zhang, Xin Wang, Weitao Yao, Zhichao Tian

**Affiliations:** ^1^ Department of Sarcoma, The Affiliated Cancer Hospital of Zhengzhou University and Henan Cancer Hospital, Zhengzhou, Henan, China; ^2^ Department of Interventional, The Affiliated Cancer Hospital of Zhengzhou University, Zhengzhou, Henan, China; ^3^ Department of Immunotherapy, The Affiliated Cancer Hospital of Zhengzhou University, Zhengzhou, Henan, China

**Keywords:** sarcoma, argon-helium knife, cryoablation, PD-1 inhibitor, immunotherapy

## Abstract

**Background:**

Effective treatment for advanced soft tissue sarcomas (STSs) is necessary for improved outcomes. Previous studies have suggested that cryoablation can have a synergistic effect with programmed cell death protein-1 (PD-1) inhibitor in the treatment of malignancy. This study aimed to clarify the efficacy and safety of argon-helium knife cryoablation in combination with PD-1 inhibitor in the treatment of STSs.

**Methods:**

Retrospectively collected and analyzed the clinical data of patients with advanced STS who underwent cryoablation and PD-1 inhibitor between March 2018 and December 2021.

**Results:**

This study included 27 patients with advanced STS. In terms of target lesions treated with cryoablation, 1 patient achieved complete response, 15 patients had partial response (PR), 10 patients had stable disease, and 1 patient had progressive disease. This corresponded to an overall response rate of 59.3% and a disease control rate of 96.3%. In terms of distant target lesions untreated with cryoablation, only two patients had a PR compared to the diameter of the lesion before ablation. The combination therapy was relatively well tolerated. None of the patients experienced treatment-related death or delayed treatment due to adverse events.

**Conclusion:**

Cryoablation combined with PD-1 inhibitors in the therapy of advanced STS is safe and can effectively shrink the cryoablation-target lesion. However, there is no evidence of the synergistic effects of this combination therapy.

## 1 Introduction

Soft tissue sarcomas (STSs) are rare heterogeneous mesenchymal tumors with more than 70 histological subtypes (1). These malignancies occur in less than 6 in 100,000 people and account for less than 2% of all adult cancers (2). Nevertheless, there are tens of thousands of new cases of STSs in China annually (3). The most important treatment for STS is surgical resection. However, more than 50% of STSs will eventually experience metastasis. Approximately 90% of advanced STS metastasizes to lung. Other common sites of metastasis include the liver, bone, and brain (4, 5). The limited availability of effective treatments for metastatic STS results in a median overall survival of 1-1.5 years for this malignancy (6, 7). Therefore, additional effective treatments are required for advanced STS.

Cryoablation is a mature technique for local tumor treatment with decades of history (8). Argon-helium knife cryoablation is performed by inserting a cryoprobe that can freeze to –150°C rapidly into the tumor and then rapidly freezing the tumor. The probe is then rapidly rewarmed to 20-40°C. Rapid temperature changes can lead to coagulation necrosis of tumor tissue within a certain range (9). Recently, cryoablation is widely used in the adjuvant treatment of various malignancies (8). Studies have preliminarily demonstrated the efficacy and safety of argon-helium knife cryoablation in the treatment of metastatic STSs (10–13). However, these studies are already outdated. In recent years, programmed cell death protein-1 (PD-1) inhibitor has been gradually used in the treatment of advanced STS (14, 15). Some studies have suggested that cryoablation combined with PD-1 inhibitor can achieve better efficacy (16, 17). However, reports on the efficacy and safety of cryoablation plus PD-1 inhibitor in STSs are rare.

As a large sarcoma center, we have significant experience in the diagnosis and treatment of sarcomas (18–20). In recent years, we have treated many patients with advanced STS with cryoablation plus PD-1 inhibitor. The clinical data of these patients were retrospectively collected and analyzed in this study, to evaluate the safety and efficacy of cryoablation plus PD-1 inhibitor in STSs. This study will provide a reference for the clinical treatment and research of advanced STSs.

## 2 Methods

### 2.1 Patients and eligibility criteria

In this retrospective study, all patients with advanced STS were treated with argon-helium knife cryoablation in combination with a PD-1 inhibitor between March 2018 and March 2022. The eligibility criteria were as follows: (1) treatment with argon-helium knife cryoablation plus a PD-1 inhibitor, (2) histologically proven STS, (3) measurable lesions, (4) multiple metastases, and (5) complete clinical data.

This study was approved by the Medical Ethics Committee of Henan Cancer Hospital (Approval No. 2021-526-002), and written consent was obtained from each patient (awareness data were collected for clinical study).

### 2.2 Treatment

Different patients were treated with different PD-1 inhibitors, including camrelizumab (Hengrui Medicine, China), sintilimab (Innovent Biologics, China), and toripalimab (Junshi Biosciences, China). The drug treatment protocols for these patients were as follows: PD-1 inhibitor was administered intravenously at a dose of 200 mg via a 30-min intravenous infusion, once every 3 weeks. Patients were treated with PD-1 inhibitor until disease progression (PD) or unacceptable adverse events (AEs).

Some patients underwent cryoablation immediately after treatment with PD-1 inhibitor; others were treated with cryoablation for better results after a period of PD-1 inhibitor treatment.

When performing cryoablation, general anesthesia and computed tomography (CT) guidance are used. A percutaneous cryoablation device utilizing 1.7-mm-diameter cryoprobe and the Cryo-HitTM cryosurgical system (CryoHit type; Galil Medical, Yokneam, Israel) are used during all procedures. The number of needles used was dependent on the size of the target lesion. Based on the size and location of the target lesion, cryoprobes were inserted into the center of the lesion mass under CT guidance. After the probe position is determined, the freeze-thaw cycle is carried out. A single cycle consists of a 10-minute freezing period during which the local temperature drops to -170°C due to the rapid expansion of argon gas, and a 2-minute rewarming period during which the local temperature rises to 20°C due to the rapid expansion of helium gas. Generally, a procedure consists of two freeze-thaw cycles. In order to obtain effective treatment, the ice hockey area needs to exceed the edge of the lesion by 1cm or more, and the scope of a single procedure needs to cover more than 80% of the lesion. After cryoablation, vital signs were monitored routinely, and hemostatic agents were administered. If there are symptoms or evidence of infection, antibiotics will be administered.

### 2.3 Evaluation

The efficacy of the treatment was evaluated using CT or magnetic resonance imaging according to the Response Evaluation Criteria in Solid Tumors version 1.1. During PD-1 inhibitor treatment, patients should be evaluated once in two treatment cycles. Patients are advised to be evaluated once a month after cryoablation. Tumor responses were categorized as complete response (CR), partial response (PR), stable disease (SD), and progressive disease (PD). The overall response rate (ORR) was defined as the sum of the CR and PR rates. The disease control rate (DCR) was defined as the sum of the ORR and SD. AEs were assessed using the National Cancer Institute Common Terminology Criteria for Adverse Events version 4.0.

### 2.4 Statistical analyses

Statistical analyses were performed using the Statistical Package for the Social Sciences (SPSS) (version 21.0, SPSS Inc., USA). Quantitative variables are presented as numerical values (percentages). The figures were drawn using GraphPad Prism 5.0 (GraphPad Software Inc., USA). This analysis was descriptive, and the follow-up period was extended to January 31, 2023.

## 3 Results

### 3.1 Patient characteristics

From March 2018 to March 2022, 27 patients with advanced STS who were treated with argon-helium knife cryoablation combined with PD-1 inhibitor were identified (**Table 1**). There were 12 men and 15 women, with an average age of 39.7 years. All patients had an Eastern Cooperative Oncology Group performance status of 0 or 1. The histological subtype included alveolar soft part sarcoma (n=5), undifferentiated pleomorphic sarcoma (n=5), leiomyosarcoma (n=3), liposarcoma (n=3), synovial sarcoma (n=3), Ewing sarcoma (n=2), fibrosarcoma (n=2), angiosarcoma (n=1), rhabdomyosarcoma (n=1), epithelioid sarcoma (n=1), and clear cell sarcoma (n=1). The lungs were the most frequent metastatic sites in most patients.

**Table 1:** Patient demographics and characteristics.

Patient No.	ECOG PS	Histological subtype	Stage	Location of target lesions	PD-1 inhibitor	Best response of the target lesions	Best response of other lesions
1	0	ASPS	IV	Lung	Sintilimab	CR	PR
2	0	ASPS	IV	Lung	Sintilimab	PR	SD
3	0	ASPS	IV	Lung	Toripalimab	SD	SD
4	1	ASPS	IV	bone	Toripalimab	SD	SD
5	1	ASPS	IV	Lung and retroperitoneum	Toripalimab	SD	SD
6	0	UPS	IV	Lung	Camrelizumab	PR	PR
7	1	UPS	IV	Lung	Sintilimab	PR	SD
8	0	UPS	IV	Head	Camrelizumab	PR	PD
9	1	UPS	IV	Bone	Sintilimab	SD	PD
10	2	UPS	IV	Lung	Camrelizumab	PD	PD
11	1	Leiomyosarcoma	IV	Lung	Sintilimab	PR	SD
12	0	Leiomyosarcoma	IV	Lung	Camrelizumab	PR	SD
13	1	Leiomyosarcoma	IV	Liver	Toripalimab	PR	PD
14	0	Liposarcoma	IV	Lung	Sintilimab	PR	SD
15	1	Liposarcoma	IV	Lung	Sintilimab	PR	PD
16	0	Liposarcoma	IV	bone	Toripalimab	SD	PD
17	0	Synovial sarcoma	IV	Lung	Camrelizumab	PR	SD
18	2	Synovial sarcoma	IV	Lung	Camrelizumab	PR	PD
19	1	Synovial sarcoma	IV	bone	Camrelizumab	SD	PD
20	0	Ewing sarcoma	IV	Lung	Sintilimab	PR	SD
21	1	Ewing sarcoma	IV	Chest wall	Sintilimab	SD	PD
22	0	Fibrosarcoma	IV	Lung	Camrelizumab	PR	SD
23	1	Fibrosarcoma	IV	Lung	Camrelizumab	SD	SD
24	1	Angiosarcoma	IV	Head	Sintilimab	SD	PD
25	1	Rhabdomyosarcoma	IV	Lung	Toripalimab	PR	PD
26	1	Epithelioid sarcoma	IV	Lung	Sintilimab	PR	PD
27	0	Clear cell sarcoma	IV	Lung	Camrelizumab	SD	PD

ECOG PS, Eastern Cooperative Oncology Group performance status; ASPS, alveolar soft part sarcoma; UPS, undifferentiated pleomorphic sarcoma; PD-1, Programmed cell death protein 1; CR, complete response; PR, partial response; SD, stable disease; PD, progressive disease.

### 3.2 Efficacy

**Figure 1:**
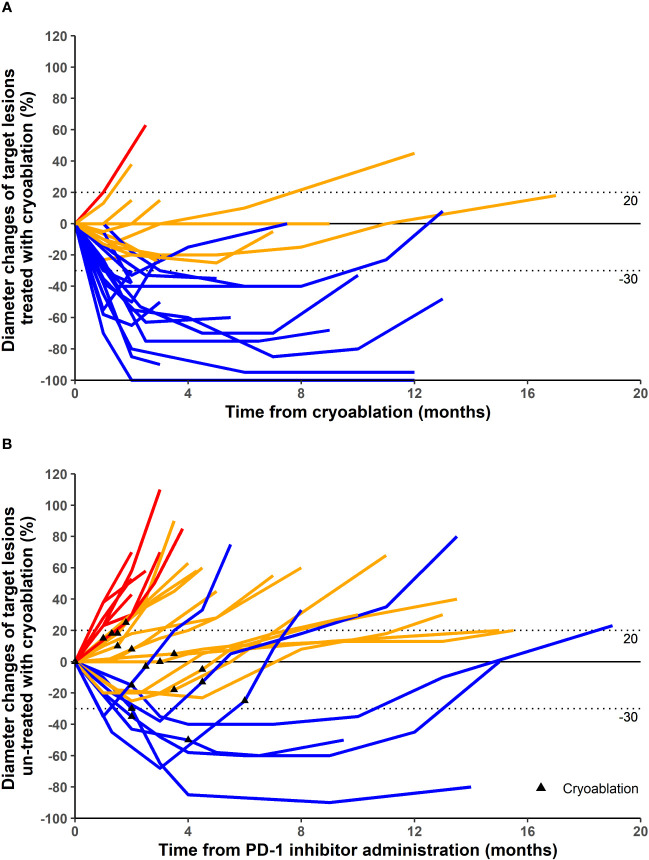
Maximum percentage diameter changes from baseline in target lesion treated with cryoablation **(A)** and untreated with cryoablation **(B)**. Treatment efficacy was evaluated using the Response Evaluation Criteria in Solid Tumors version 1.1. **(A)** The baseline is the diameter of target lesions before cryoablation. **(B)** The baseline is the diameter of distant lesions (untreated with cryoablation) before the first dose of programmed cell death protein 1 (PD-1) inhibitor. Some patients underwent cryoablation immediately after treatment with PD-1 inhibitor, and others were treated with cryoablation for better results after a period of PD-1 inhibitor treatment.

**Figure 2:**
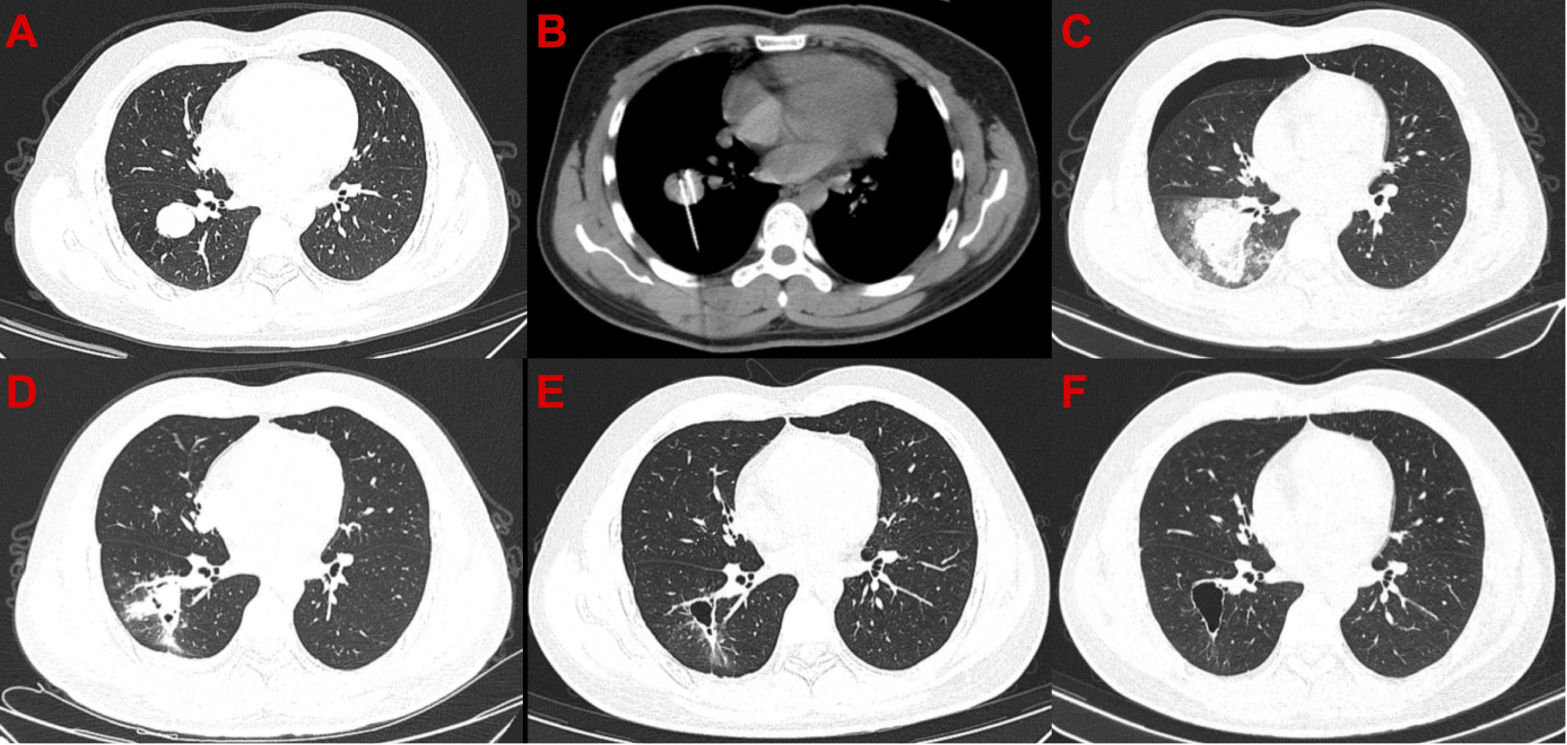
Computed tomography images of the only patient with complete response after cryoablation. **(A)** Before cryoablation. **(B)** During cryoablation. **(C)** After cryoablation, local large-scale inflammation and pneumothorax can be observed. **(D)** One month after cryoablation, pneumothorax disappeared, and the scope of inflammation narrowed. **(E)** Moreover, 3.5 months after cryoablation, cavity formation was observed in the diseased area. **(F)** 7.5 months after cryoablation.

Different patients were treated with different PD-1 inhibitors. A total of three PD-1 inhibitors were used (**Table 1**). In terms of target lesions treated with cryoablation, 1, 15, 10, and 1 patients had CR, PR, SD, and PD, respectively (**Table 1**; **Figures 1**, **2**). This corresponded to an ORR of 59.3% and a DCR of 96.3%. In terms of distant target lesions untreated with cryoablation, only two patients had a PR compared to the diameter of the lesion before ablation (**Table 1**; **Figure 1**).

### 3.3 Safety

**Figure 3:**
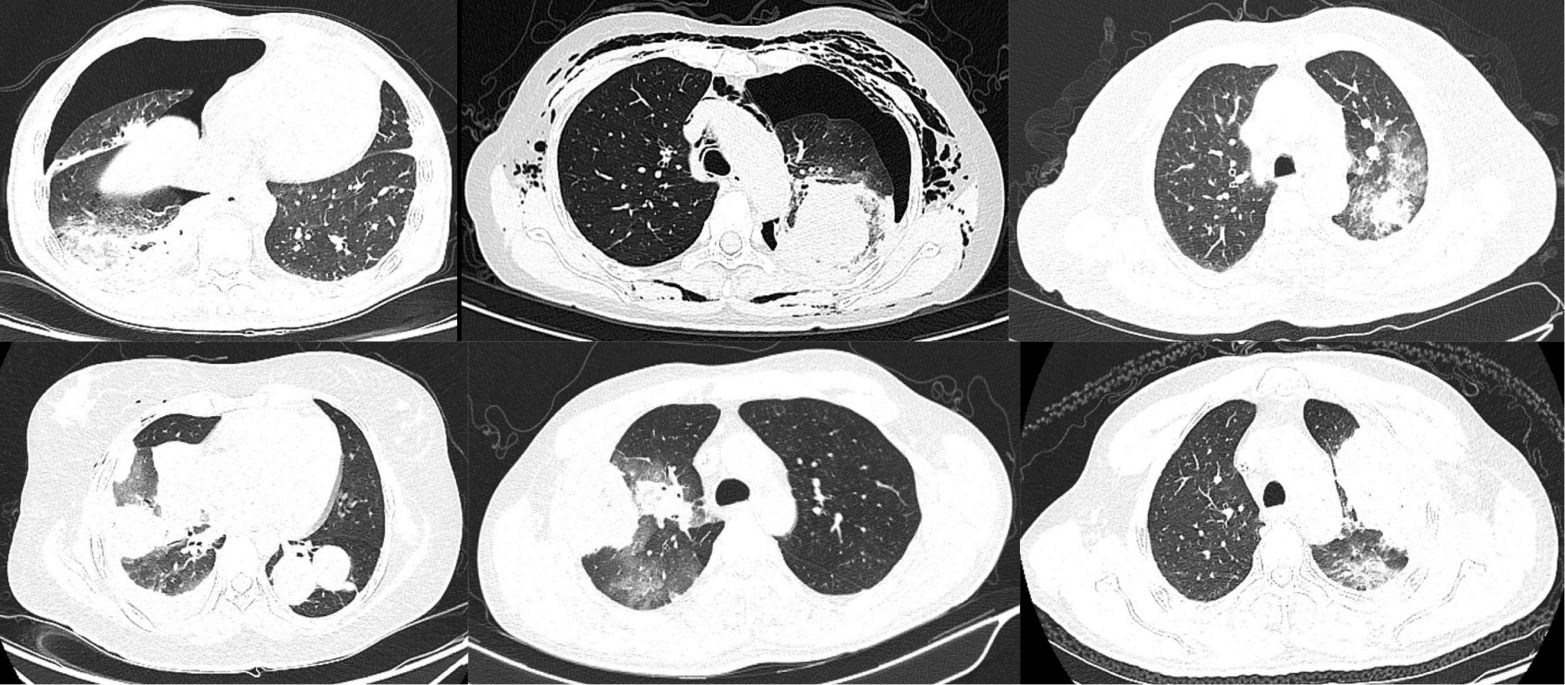
Lung inflammation after cryoablation. Nearly all lung lesions developed local pneumonia after cryoablation, and some patients also had pneumothorax. All these inflammations disappeared within 1 month after cryoablation.

In general, argon-helium knife cryoablation combined with PD-1 inhibitor therapy was relatively tolerated (**Table 2**). Grade 1 or 2 AEs associated with cryoablation included the following: pneumonitis (63.0%) (**Figures 2**, **3**), fever (55.6%), pneumothorax (40.7%), pleural effusion (29.6%), cough (25.9%), pain (18.5%), and nerve injury (3.7%). Grade 1 or 2 AEs associated with PD-1 inhibitor included the following: hypothyroidism (18.5%), fatigue (14.8%), increased transaminase increase level (11.1%), pneumonitis (11.1%), rash (11.1%), and diarrhea (7.4%). Grade 3-4 AEs were rare. None of the patients experienced delayed treatment or treatment-related death due to AEs.

**Table 2:** Adverse events.

Adverse events (AEs)	Grade 1-2	Grade 3-4
AEs related to cryoablation		
Pneumonitis	63.0% (17/27)	11.1% (3/27)
Fever	55.6% (15/27)	
Pneumothorax	40.7% (11/27)	3.7% (1/27)
Pleural effusion	29.6% (8/27)	3.7% (1/27)
Cough	25.9% (7/27)	3.7% (1/27)
Pain	18.5% (5/27)	
Nerve injury	3.7% (1/27)	3.7% (1/27)
AEs related to immunotherapy		
Hypothyroidism	18.5% (5/27)	3.7% (1/27)
Fatigue	14.8% (4/27)	3.7% (1/27)
Transaminase increase	11.1% (3/27)	
Pneumonitis	11.1% (3/27)	
Rash	11.1% (3/27)	
Diarrhea	7.4% (2/27)	

Data are presented as percentages (number events/total).

## 4 Discussion

Although there are dozens of subtypes of STSs, most are immunotherapeutic indolent. The results of one of the most influential clinical trials reported in 2017 showed that only 7 of the 40 patients with STS had an objective response (21). Many other studies have found similar results (15). To improve the efficacy of STS immunotherapy, various approaches are now being explored to enhance the immunogenicity of sarcomas (22, 23). Previous studies have suggested tissue and cell destruction caused by cryoablation may increase the expression of tumor antigens, thereby enhancing the ability of the immune system to recognize and attack tumor cells (24, 25). Currently, only one study has reported the safety of cryoablation combined with PD-1 inhibitor therapy in STSs, but it has not shown significant synergistic efficacy (16).

In this study, 27 patients with STS were received argon-helium knife cryoablation plus PD-1 inhibitor treatment. The results showed that 96.3% (26/27) of the patients experienced the control of the size of target lesions. This suggests that cryoablation is effective in treating STS metastases. However, only 7.4% (2/27) of the patients receiving cryoablation showed a reduction in the diameter of distant lesions. This suggests that cryoablation combined with PD-1 inhibitor treatment is hard to produce synergistic effects as it does in other cancers (26, 27). There are many factors affecting the efficacy of PD-1 inhibitor. First, it may be due to the immune inertia of the sarcoma itself that the local increase in immunogenicity produced by cryoablation is insufficient to activate the body’s systemic antitumor immune response. Second, cryoablation inevitably leads to local bleeding, necrosis, and inflammatory reaction (**Figures 2**, **3**). Nevertheless, the inflammatory response inhibits the efficacy of PD-1 inhibitor (28). Therefore, the effect of cryoablation on antitumor immune response is two-sided. It may improve antitumor immune response by improving immunogenicity or inhibit antitumor immune response by triggering inflammatory response. A study that found that the freezing rate of cryoablation had a significantly different effect on the immune system may confirm our conjecture (29). Third, adjuvant medication has an important effect. A prior study confirmed that the use of concomitant drugs (steroids, systemic antibiotics, proton pump inhibitors) was associated with worse clinical outcomes when receiving PD-1 inhibitor (30). In this study, we used the abovementioned adjuvant therapy agents in almost every patient. In addition, the combination sequence of cryoablation with PD-1 inhibitors may also be important. A current study has demonstrated that different sequences of chemotherapy combined with PD-1 inhibitors have significantly different effects on efficacy (31). In this study, all patients were treated with PD-1 inhibitor before cryoablation. This combination sequence may not be conducive to synergistic efficacy. A systematic follow-up study for a valid comparison of PD-1 inhibitor treatment before and after cryoablation can demonstrate which strategy could be clinically beneficial. In conclusion, there are many factors that lead to the lack of synergistic effect between cryoablation and PD-1 inhibitors in this study, and further studies are required. Additionally, it is worth noting that some studies have demonstrated that thermal ablation can promote the release of tumor antigens, thereby driving downstream immune responses (32–34). The differences in the effects of different ablation methods such as thermal ablation or cryoablation on anti-tumor immunity are also worth further research.

In general, cryoablation combined with PD-1 inhibitor has a safety profile, with rare serious complications. The most common complications are pneumonia and pleural effusion after treatment of lung lesions (**Figures 2**, **3**). Patients with these complications are often treated with adjuvant medications, such as steroid hormones or antibiotics. Such adjunctive agents inhibit the antitumor immune response and even lead to a poor prognosis (30). This may be one reason for the absence of synergistic results in this study. Therefore, PD-1 inhibitor should not be considered in patients considering lung cryoablation until steroid hormones or antibiotics are confirmed to be unnecessary.

We acknowledge that the small sample size, retrospective nature, and lack of a control group lower the level of evidence in this study. Although cryoablation does not activate the antitumor immune response, it shrinks the cryoablation-target lesions and improves patients’ symptoms, which may be beneficial in prolonging the survival of patients with advanced STS with insufficient effective treatment. Therefore, cryoablation remains an important therapeutic treatment for some advanced STSs, and the interaction mechanism between cryoablation and immunotherapy should be further studied in the future.

In conclusion, cryoablation combined with PD-1 inhibitors in the treatment of advanced STS is safe and can effectively shrink the cryoablation-target lesion. However, there is no evidence of the synergistic effects of this combination therapy.

## Data availability statement

The original contributions presented in the study are included in the article/supplementary material. Further inquiries can be directed to the corresponding author.

## Ethics statement

The studies involving humans were approved by Medical Ethics Committee of Henan Cancer Hospital. The studies were conducted in accordance with the local legislation and institutional requirements. The participants provided their written informed consent to participate in this study. The manuscript presents research on animals that do not require ethical approval for their study.

## Author contributions

JW and ZT were responsible for the conception, design and drafting of the study. DZ, SD, SG, PZ, XW and WY were responsible for the patient recruitment and clinical investigation. JW, YY, WY and ZT were responsible for the analysis and interpretation of data. All authors contributed to the article and approved the submitted version.
